# Fluconazole Resistance and Virulence in In Vitro Induced-Fluconazole Resistant Strains and in Clinical Fluconazole Resistant Strain of *Cryptococcus deuterogattii*

**DOI:** 10.3390/pathogens12060758

**Published:** 2023-05-24

**Authors:** Sébastien Bertout, Laetitia Laroche, Frédéric Roger, Donika Krasteva, Pascal Drakulovski, Virginie Bellet

**Affiliations:** 1 Laboratoire de Parasitologie et Mycologie Médicale, TransVIHMI, University of Montpellier, INSERM, IRD, 15 Avenue Charles Flahaut, 34093 Montpellier, France; sebastien.bertout@umontpellier.fr (S.B.); frederic.roger1@umontpellier.fr (F.R.); donika.krasteva@umontpellier.fr (D.K.); pascal.drakulovski@umontpellier.fr (P.D.); 2 Laboratoire de Biologie Médicale, Hôpital Lozère, 48000 Mende, France; laetitialaroche@ch-mende.fr

**Keywords:** fluconazole, resistance, *Cryptococcus gattii* species complex, *Cryptococcus deuterogattii*, MDR1, ERG11, AFR1, AFR2, virulence, *Galleria mellonella*

## Abstract

Neuromeningeal cryptococcosis is a life-threatening infection of the central nervous system, caused by encapsulated yeast belonging to the *Cryptococcus neoformans* and *Cryptococcus gattii* species complexes. Recent data showed that virulence and antifungal resistance are variable for yeasts belonging to the *C. gattii* species complex. There is an increase in resistance to fluconazole for yeasts of the *C. gattii* species complex and the virulence is variable according to the genotype. In the present study, (i) we explored and compared the mechanisms of resistance to fluconazole between *C. deuterogattii* clinically resistant strains and induced fluconazole-resistant strains by exposure to fluconazole in vitro, and (ii) we studied their virulence in the *Galleria mellonella* study model. We demonstrated that the fluconazole resistance mechanisms involved were different between clinically resistant strains and induced resistant strains. We also demonstrated that fluconazole-induced resistant strains are less virulent when compared to the original susceptible strains. On the contrary, the clinically resistant strain tested maintains its virulence compared to fluconazole-susceptible strains of the same sequence type.

## 1. Introduction

Neuromeningeal cryptococcosis is an infection of the central nervous system resulting in an estimated 152,000 cases of meningitis and 112,000 deaths annually worldwide, 75% of which occur in sub-Saharan Africa [1,2,3]. In sub-Saharan Africa, it is the second most common cause of meningitis and death from opportunistic infections in people living with HIV (PLHIV) [4,5]. Recent data have shown a reduction in the estimated absolute global burden of HIV-associated cryptococcal meningitis compared with 2014 [2], likely to be due to antiretroviral therapy expansion [1]. Inhalation of the spores leads to a primary pulmonary infection that is often asymptomatic. Following this, various clinical forms may occur [6]. Neuromeningeal cryptococcosis is the most frequent form and mainly manifests as headaches, fever, dizziness, behavioural disorders, seizures, obnubilation and even coma [7]. Without treatment, this clinical form evolves, possibly leading to the death of the patient. Despite treatment, the risk of death in PLHIV remains approximately 20 to 30% in developed countries and can reach 70% in developing countries [3,8,9]. The causative agent of cryptococcosis is an encapsulated yeast belonging to the *Cryptococcus neoformans* and *Cryptococcus gattii* species complexes [10,11]. These fungi occur naturally in the environment. The taxonomy of the *Cryptococcus* species complexes was revisited first in 2016 [11] and more recently in 2021 [12]: a consensus seems to be accepted around the *C. neoformans* species complex subdivided into *C. neoformans* and *C. deneoformans* and the *C. gattii* species complex subdivided into *C. gattii* (serotype B; genotype VGI), *C. bacillisporus* (B and C; VGIII), *C. deuterogattii* (B; VGII), *C. tetragattii* (C; VGIV) and *C. decagattii* (B; VGVI). Three hybrids also exist. These two complexes differ in terms of biotope [13,14] but also clinically. Indeed, yeasts of the *C. gattii* species complex affect both immunocompromised and immunocompetent individuals, whereas yeasts of the *C. neoformans* species complex mainly infect immunocompromised patients [15]. The neuromeningeal infections caused by strains from the *C. gattii* species complex are also more severe and often present with numerous complications, such as asymptomatic pulmonary nodules or mass lesions named cryptococcomas [16,17]. The therapeutic regimen available to treat cryptococcal infections is relatively limited. The consensus treatment protocol published in 2018 by the World Health Organization is based on induction therapy with amphotericin B (AMB) combined with 5-fluorocytosine (5FC) for seven days followed by fluconazole (FCZ) 1200 mg/d for one week. Consolidation treatment is based on FCZ 800 mg/d for eight weeks [3]. In low-income countries, especially in sub-Saharan Africa, only FCZ is widely available and is used as monotherapy throughout treatment in more than 80% of cases [17], with AMB and 5FC rarely available [18,19].

Minimum inhibitory concentrations (MICs) to azoles tend to be higher for yeasts of the *C. gattii* species complex, mainly for *C. deuterogattii* strains, than for *C. neoformans* [20,21]. The cryptococcal relapses observed in South Africa could be due to treatment failure following these high MICs [22]. Increasing the length and dose of antifungal treatment seems necessary in cases of infection with yeasts of this complex species [23]. Regarding antifungal cryptococcal resistance, recent data in the literature shows an increase in resistance, particularly to FCZ, throughout the world [24,25], especially in sub-Saharan Africa [26]. According to a systematic review, this region has the highest rate of resistance to FCZ, with approximately between 43.6 and 50% of strains being resistant [27]. There is a difference in virulence between the two *Cryptococcus* species complexes. Indeed, *C. neoformans* develops mainly in immunocompromised individuals, thus requiring the host’s permissiveness, unlike *C. gattii*, which can also affect immunocompetent individuals [28]. There is still very little data on the virulence of resistant strains. A study on *C. neoformans* strains from Spain suggests that virulence is not dependent on resistance to FCZ but mainly on genotype [29]. Another study showed that genetically similar strains could show different virulence potential [30]. In previous work, all clinical strains of *C. deuterogattii* from Côte d’Ivoire were found through multilocus sequence typing analysis only from ST 173 [31]. In terms of the mechanisms of resistance to FCZ, no mutations in the ERG11 gene of resistant clinical strains have been found, and only overexpression of the AFR1 efflux pump has been demonstrated in these strains upon exposure to FCZ [32].

The objectives of this study are (i) to further investigate the mechanisms of resistance to FCZ in African clinical *Cryptococcus deuterogattii* ST173 strains, (ii) to compare the resistance mechanisms of in vitro induced FCZ-resistant strains of *Cryptococcus deuterogattii* by exposure to fluconazole and (iii) to characterise the virulence of the African strains in the *Galleria mellonella* study model and thus visualise the link between virulence and resistance.

## 2. Materials and Methods

### 2.1. Strains and Growth Media

We used FCZ-susceptible clinical strains: three Ivorian strains of *C. deuterogattii* ST 173 which were isolated from the cerebrospinal fluid (CSF) of three Ivorian patients that we previously described [32,33] named Cg1, Cg2 and Cg3, and one reference strain R265 (CBS10514), ST20. A total of five and three Induced FCZ-resistant (“IR”) strains of *C. deuterogattii* ST173 (named Cg1-IR_1,_ Cg1-IR_2,_ Cg1-IR_3,_ Cg1-IR_4_ and Cg1-IR_5_) and *C. deuterogattii* R265 ST20 (named R265-IR_1,_ R265-IR_3_ and R265-IR_5_), respectively, were tested in this study. The five IR strains of *Cryptococcus deuterogattii* ST173 were obtained from only one Ivorian strain of *C. deuterogattii* ST 173 (Cg1). IR FCZ-strains were obtained by growing FCZ-susceptible strains (R265 or Cg1) on yeast extract peptone dextrose (YEPD) liquid medium supplemented with increasing concentrations of FCZ, starting at half the MIC to FCZ value. After 72 h, 10 µL of this suspension was inoculated on YEPD supplemented with FCZ at the MIC concentration. The process was repeated 72 h later, with strains growing in a stepwise manner, at successively increasing concentrations of FCZ, up to the value at which growth ceased. The susceptibility to antifungals of all the isolates was tested using the reference broth microdilution method of the CLSI (Clinical and Laboratory Standards Institute). The MIC to FCZ was 16 and 8 µg/mL, respectively, for the R265 and Cg 1 strains. The MIC to FCZ was 8 and 2 µg/mL for the Cg 2 and Cg 3 strains, respectively. All the IR strains and the clinical FCZ resistant strain (Cg R) [32] have a MIC to FCZ equal to 64 µg/mL. All the IR FCZ strains are then grown on Sabouraud chloramphenicol agar medium supplemented with 64 µg/mL of FCZ, for all experiments.

We used the two quality control strains *C. krusei* ATCC 6258 and *C. parapsilosis* ATCC 22,019 for *xxx* susceptibility to antifungals testing as recommended by the CLSI.

For the virulence study on *Galleria mellonella,* we used three strains with known virulence as controls strains *C. deuterogattii* ST 7, *C. gattii sensu stricto* ST 106 and *C. deuterogattii* ST 20 (R265). Both strains of *C. deuterogattii* were isolating during the Vancouver outbreak.

The strains, except the resistant or IR strains, were grown on Sabouraud chloramphenicol agar. RPMI medium was used for antifungal microdilution susceptibility testing.

### 2.2. Antifungal Susceptibility Testing

The in vitro susceptibility profile of *Cryptococcus deuterogattii* against FCZ was determined using the reference broth microdilution method in accordance with the CLSI. The final antifungal concentrations ranged from 0.25 to 64 μg/mL for fluconazole. The minimal inhibitory concentrations (MICs) for FCZ were defined as concentrations causing a 50% reduction in turbidity compared to the growth of the control at 72 h. *Candida krusei* ATCC 6258 and *Candida parapsilosis* ATCC 22,019 were used as control strains [34].

With no breakpoint values, we use epidemiological cut-off values (ECVs) to discriminate susceptible strains from resistant strains with reduced susceptibility to some antifungals.

### 2.3. RNA Isolation, Reverse Transcription, and Real-Time Quantitative PCR Analysis

For RNA extraction, cells were grown overnight in 10 mL of YEPD medium (supplemented with 64 µg/mL of FCZ for IR strains) on a shaker at 30 °C. Following 24 h of growth, cells were harvested by centrifugation to obtain 1 × 10^8^ cells. Total RNA was extracted using the Ambion RiboPure-Yeast extraction kit^®^ (Thermo Fisher Scientific, Waltham, MA, USA), following the manufacturer’s recommendations for RNA isolation. To remove genomic DNA contamination, RNA samples were treated with DNAse. After extraction, RNA was quantified using a Nanodrop 2000 UV-visible spectrophotometer (Thermo Scientific) and the quality of purified RNA was assessed on an Agilent 2100 bioanalyzer (Agilent Technologies, Santa Clara, CA, USA).

cDNA was synthesized using the SuperScript IV First-Strand Synthesis system (Invitrogen), followed by Real-time quantitative PCR with a LightCycler1 480 Real-Time PCR Instrument (Roche). Forward and reverse primers sequences for ERG11, MDR1, AFR1, AFR2 and ACTIN [32] were used at a 57 °C annealing temperature. Data were normalized to actin gene levels and were expressed for IR strains as the amount of mRNA relative to that of strain R265, ST20 or *C. deuterogattii* ST173.

### 2.4. DNA Extraction and ERG11 Sequencing

Genomic DNA was extracted for each strain using extraction kit NucleoSpin blood quick (Macherey-Nagel Gmb and Co. KG, Duren, Germany) with modifications as previously described [30]. The whole ERG11 gene was amplified by PCR as follows: initial denaturation (94 °C, 5 min), 35 cycles of denaturation (94 °C, 1 min), annealing (57 °C, 30 s) and extension (72 °C, 1 min) and a final extension cycle (72 °C for 10 min) with specific primers for *C. deuterogattii* as previously described [33].

Amplicons were purified and sequenced using the four primers for *C. deuterogattii* strains to sequence the whole ERG11 gene by Genewiz, Leipzig, Germany. Sequences were manually edited and aligned using BioEdit software.

### 2.5. Survival Curve of *Galleria mellonella* Infected with *Cryptococcus Strains*

*G. mellonella* in the final larval stage were stored in the dark before use. Insect rearing has been conducted in the DGIMI’s Quarantine insect facility, Faculty of Sciences of Montpellier. Ten randomly chosen larvae with similar weight (200 mg) and size were used per group in all assays. Two control groups were included: one was inoculated with PBS to observe the killing due to physical trauma, and the other received no injection to determine baseline viability. *Cryptococcus deuterogattii* strain ST20 and ST173 were previously cultured in sabouraud medium at 37 °C for 24 h. Yeast cells were counted, and a solution of 10^8^ yeasts/mL were prepared in PBS and 20 μL were injected into the hemocell of larvae via the last left proleg containing 2 × 10^6^ cells/larvae or PBS for control group. After injections, larvae were incubated in Petri dishes at 37 °C and monitored for survival daily from day 3 to day 12.

The virulence of the three ST173 FCZ susceptible strains was evaluated. In a second time, for the IR strains, only one resistant strain of R265 and Cg1 were used. These strains were considered as representative of all other isolates.

### 2.6. Statistical Analysis

An unpaired two-tailed Student’s *t*-test was used to compare the fold change values of the R265 strain vs. all the R265-IR strains (A) and Cg1 vs. all the Cg1-IR strains (B). The statistical significance is shown with an asterisk: * *p*-value < 0.05. The absence of an asterisk indicates that the fold change values are not significant.

Survival in *G. mellonella* infection studies was compared with the log-rank (Mantel–Cox) test using GraphPad Prism software.

## 3. Results

### 3.1. No Mutations Are Detected in the ERG 11 Gene in Any of the Induced FCZ-Resistant Strains

The Ivorian *C. deuterogattii* ST173 strain and its five IR strains showed one difference in the ERG11 gene compared to the *C. deuterogattii* R265 ST20 strain and its three IR strains. This mutation (C101A) led to a substitution of histidine at position 50 with asparagine (H50N). However, no other mutation in ERG11 appeared in any of the IR strains compared to their corresponding FCZ-susceptible strain.

### 3.2. ERG11, MDR1, AFR1 and AFR2 mRNA Expression

**Figure 1:**
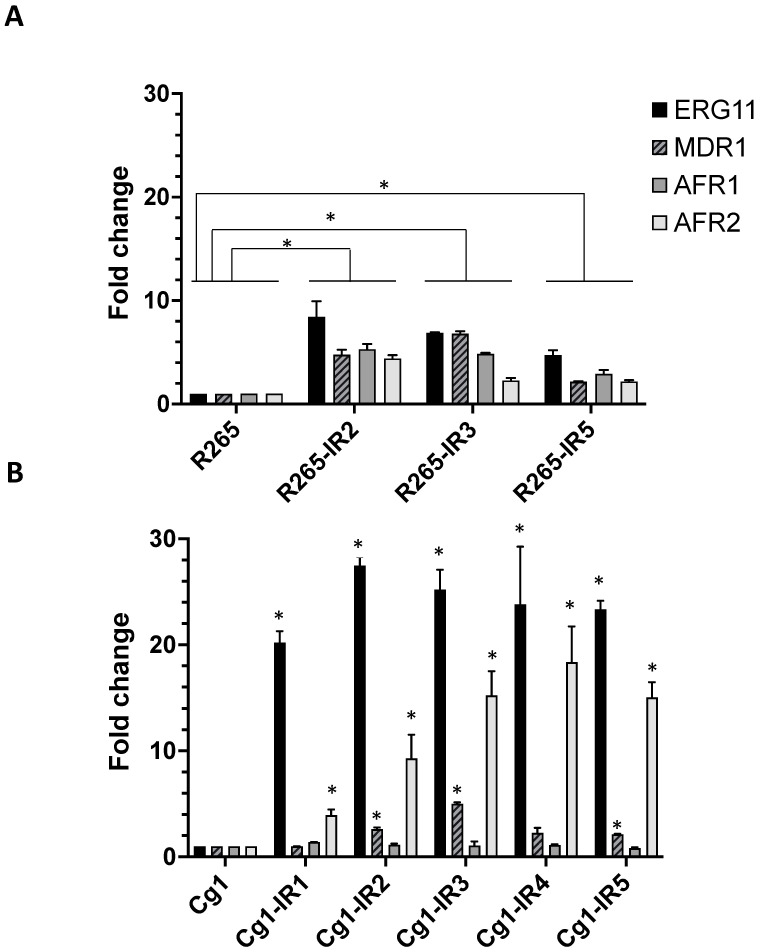
Expression rate of the different genes in studied strains. (**A**) In the reference strain *C. deuterogattii* ST20 (R265) and 3 induced fluconazole-resistant strains: R265-IR2, R265-IR3 and R265-IR5; (**B**) In *C. deuterogattii* ST173 strain (Cg1) and 5 induced fluconazole-resistant strains: Cg1-IR1, Cg1-IR2, Cg1-IR3, Cg1-IR4 and Cg1-IR5. Values represent the mean ± standard deviation of three biological replicates. The statistical significance is shown with an asterisk: * *p* < 0.05. The absence of an asterisk indicates that the fold change values are not significant.

We studied mRNA expression of ERG11, MDR1, AFR1 and AFR2 genes in the *C. deuterogattii* R265 ST20 strain and the three following IR strains (Figure 1A), as well as in the Ivorian *C. deuterogattii* ST173 strain and the five following IR strains (Figure 1B). Fold changes were obtained by comparing the ratio (gene of interest/housekeeping gene) for each strain with the ratio of the reference strain R265 or Ivorian fluconazole-susceptible *C. deuterogattii* strain.

*C. deuterogattii* R265-induced FCZ-resistant strains showed overexpression of ERG11 (average fold change 6.68 ± 1.86). The three efflux pumps, MDR1, AFR1 and AFR2, were also overexpressed (average fold change 4.58 ± 2.32; 4.36 ± 1.27; 2.95 ± 1.26, respectively). (Figure 1A). The overexpression of ERG11, MDR1, AFR1 and AFR2 is significant between the three FCZ IR strains and the R265 strain.

All five *C. deuterogattii* ST173-induced fluconazole-resistant strains showed overexpression of ERG11 and AFR2 efflux pumps (average fold changes of 24.0 ± 2.67 and 12.38 ± 5.74, respectively) compared to the Cg1 strain. MDR1 was overexpressed but less than ERG11 and AFR2 (average fold change 2.6 ± 1.47) and the overexpression was not significant for all the IR strains. AFR1 was not overexpressed (average fold change 1.11 ± 0.20) as compared to the Cg1 strain.

### 3.3. Virulence Studies on G. melonella Larvae

#### 3.3.1. Virulence Study of Ivorian *C. deuterogattii* Strains

**Figure 2:**
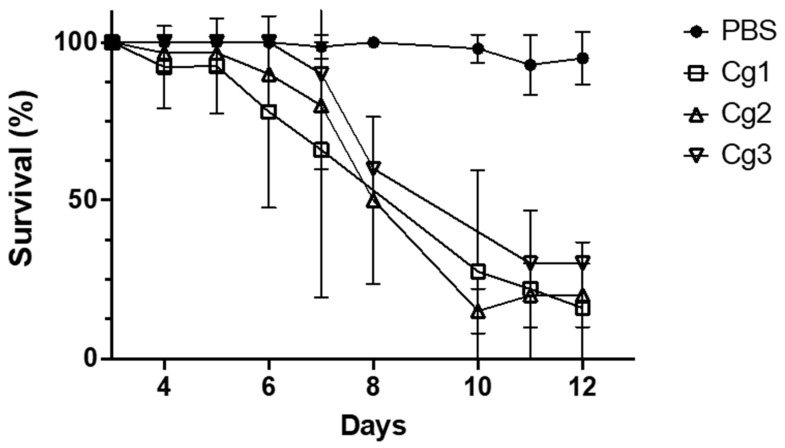
Virulence of three Ivorian strains of *C. deuterogattii* ST 173 in *G. mellonella.* Values represent the mean ± standard deviation of three biological replicates.

To study Ivorian *C. deuterogattii* strain virulence, we first compared virulence from three Ivorian *C. deuterogattii* strains named Cg1, Cg2 and Cg3, which were isolated from three patients. Comparable survival curves were obtained (Figure 2). Therefore, for all the experiments, we used only the Cg1 strain.

**Figure 3:**
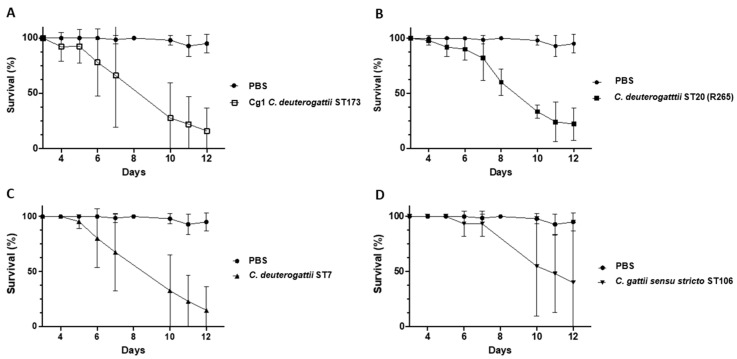
Virulence of Cg1 (**A**), R265 (**B**), *C. deuterogattii* ST7 (**C**) and *C. gattii sensu stricto* ST106 strains (**D**) in *G. mellonella*. Values represent the mean ± standard deviation of three biological replicates.

The virulence of the Ivorian Cg1 strain was then compared with the virulence of three other strains for which virulence has already been described ((Figure 3): the reference strain *C. deuterogattii* ST 20 (R265), *C. deuterogattii* ST 7 and *C. gattii sensu stricto* ST106).

At D12 post-injection, the average survival was 20% ± 0.22 for larvae infected by the Cg1 strain (Figure 3A) and 23% ± 0.15 for larvae infected by the R265 strain (Figure 3B). For the strain *C. deuterogattii* ST 7, it was 15% ± 0.21 (Figure 3C), and for the strain *C. gattii sensu stricto* ST106, it was 40% ± 0.56 (Figure 3D). The Cg1 strain is as virulent as the *C. deuterogattii* strains (R265 or *C. deuterogattii* ST 7). The Cg1 isolates showed virulence comparable to the two strains from the Vancouver outbreak.

#### 3.3.2. Study of the Relationship between Virulence and Resistance

**Figure 4:**
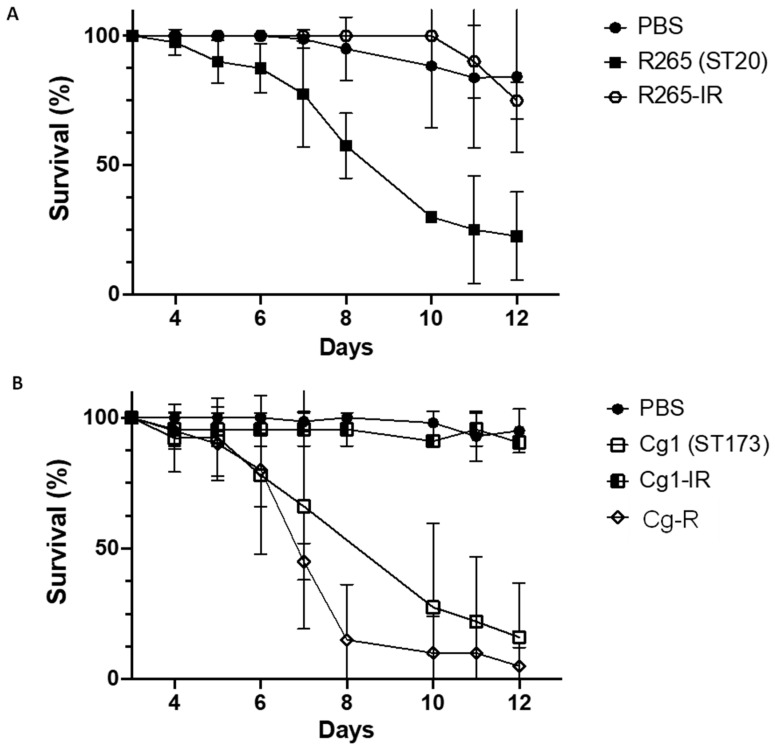
Virulence of strains in *G. mellonella*. (**A**) Survival curve for the *C. deuterogattii* ST20 strains (R265, R265-IR); (**B**) Survival curve for the *C. deuterogattii* ST173 strains (Cg1, Cg1-IR, Cg-R). Values represent the mean ± standard deviation of three biological replicates.

To determine the potential relationship between virulence and resistance, we compared the virulence of Cg1 and R265 strains with their induced fluconazole-resistant strains (Cg1-IR and R265-IR) and with a fluconazole-resistant strain isolated from a patient (Cg-R) (Figure 4).

The average survival was 75% ± 0.07 for the R265-IR strain (Figure 4A) and 91% ± 0.01 for the Cg1-IR strain, whereas it was 27% ± 0.38 for Cg-R (Figure 4B) at D12. The average survival for the Cg1 strain was 20% ± 0.22 at D12 (Figure 4B). R265-IR and Cg1-IR are no longer virulent as their survival curves are comparable to the control survival curve (PBS). On the contrary, the survival curve of the Cg-R strain is comparable to the survival curve of the Cg1 strain.

## 4. Discussion

Within the *C. gattii* species complex, the ECVs that classify strains as wild-type or mutant to FCZ are highly variable depending on serogenotype. Strains, except *C. deuterogattii*, are considered resistant when their MIC to FCZ is greater than 8 μg/mL. *C. deuterogattii* is considered resistant when its MIC to FCZ is strictly above 32 μg/mL [34]. Strains of the *C. gattii* species complex therefore generally have higher MICs to FCZ than average MIC values to FCZ [32,33], raising questions about the origin of this increase. To try to understand the mechanisms by which the *C. deuterogattii* strain increases its MIC to FCZ, we worked on clinical *C. deuterogattii* strains resistant and susceptible to FCZ. We have previously worked to try to elucidate the mechanisms of resistance to FCZ in *Cryptococcus* strains ST 173 [32]. *Cryptococcus deuterogattii* ST 173 was first described in 2012 [35] and then in 2016 because it was isolated from the CSF of Ivorian PLHIV with neuromeningeal cryptococcosis [31,32]. This ST was poorly described and studied. In our previous work, on 28 Ivorian *C. deuterogattii* ST 173 strains, 64.3% had a high MIC to FCZ (≥16 μg/mL) [31,32]. We have demonstrated that these strains exhibited no mutations in the ERG11 gene which could explain the increase in FCZ MICs [32]. Strains had increased ERG11 and MDR1 mRNA expression, while AFR1 and AFR2 were not overexpressed in strains with high FCZ MICs compared to the expression levels for strains with low FCZ MICs. Exposure to FCZ in strains with high MICs induced AFR1 mRNA overexpression. Here, we studied the gene expression rates of ERG11, MDR1, AFR1 and AFR2 in the *C. deuterogattii* R265 strain and its three induced fluconazole-resistant strains and in the Ivorian *C. deuterogattii* strain and its five induced fluconazole-resistant strains. These four genes are commonly described as involved in FCZ resistance. We observed overexpression of ERG11 and MDR1 in induced fluconazole-resistant strains. Concerning AFR1 and AFR2, AFR1 was overexpressed in R265-induced fluconazole-resistant strains, and AFR2 was overexpressed in Ivorian *C. deuterogattii*-induced fluconazole-resistant strains. The mechanism leading to fluconazole resistance in strains exposed to fluconazole in vitro appears to differ depending on the ST for the same species. In the literature, no study has compared resistance and molecular response to fluconazole exposure in relation to ST, but it has sometimes been reported that the susceptibility to FCZ varies depending on the molecular type [36]. However, in the fluconazole-resistant clinical strain, no overexpression of these four genes appears to be the cause of fluconazole resistance, as confirmed by a previous study [32]. According to the literature, the overexpression of PDR11 (AFR1 homologue in *C. neoformans*) is thought to be the cause of fluconazole resistance in *C. deuterogattii* from the Vancouver outbreak [37]. AFR1 was described as the major drug efflux pump. Our results are in agreement with these data from the literature since the R265 strain was the predominant genotype found during the Vancouver outbreak. In contrast, the unique involvement of AFR2 in fluconazole resistance has never been described before in *C. gattii* strains. However, it has been only previously demonstrated that if AFR2 and MDR1 were deleted in combination with AFR1, a minor additive effect in susceptibility toward several azoles was observed [38]. Our results suggest that it is the differential expression of the two efflux pumps AFR1 and AFR2 that may vary according to the type sequence of the strain. This will have to be confirmed on different ST strains within the C. *deuterogattii* genotype. It is also unclear whether this is true for other genotypes within the *C. gattii* species complex. The identification of other genes or mechanisms involved in FCZ resistance in *Cryptococcus gattii* is necessary to understand the increase in FCZ resistance in these strains but also to understand the particular behavior of the VGII genotype towards fluconazole resistance. Recent data indicate that a novel ABC pump Afr3, was upregulated in *C. neoformans* cells and may contribute to their enhanced FCZ tolerance, by promoting drug efflux [39]. A new study revealed the importance of fungal PDR-type transporters in *Cryptococcus neoformans* by the study of PDR6. An altered antifungal susceptibility profile, including hypersensitivity to FCZ were shown in a strain of *C. neoformans* with this gene deleted [40]. No study has focused on these two genes in *C. gattii*. It might be interesting to study them in the strains of this complex species. As a second step, the objective of our study was to study the virulence of *C. deuterogattii* ST 173 from Côte d’Ivoire, from both susceptible and resistant strains to fluconazole, on an experimental model of *Galleria mellonella* insect larvae. According to the literature, the virulence of *Cryptococcus* varies according to serotype and genotype [41,42]. Concerning the *C. gattii* complex species, some authors have said that the pathogenicity of *Cryptococcus* strains in the *G. mellonella* invertebrate model is independent of molecular type or pathogenicity factor, even within the same ST, but it is possible to find variable degrees of pathogenicity [38]. The most representative example is the strains from the Vancouver outbreak which are *C. deuterogattii* strains, genotype VGII but of different ST, and they have different virulence [43,44]. In our study, all the *C. deuterogattii* strains tested have a similar virulence in contrast to the *C. gattii sensu stricto* strain, the same serotype B, but are of a different genotype which is less virulent in this experimental model. Our results are therefore at odds with the literature, but we confirmed that virulence could depend on genotype for strains of the *C. gattii* complex species. We also, thereafter, compared the virulence of induced fluconazole-resistant strains with a fluconazole-resistant clinical strain isolated from one patient. We have shown that in *C. deuterogattii* strains, exposure to fluconazole to render them resistant makes the strains less virulent. This is observed for the two tested strains of different ST. Generally, virulence decreases with the development of resistance to antimicrobial agents [45]. This has been demonstrated in bacteria [46] but also in yeast, such as *Candida* species [47]. This was previously observed for *C. neoformans* and *C. gattii* strains, showing that the adaptation of isolates to selective antifungal pressure influenced the loss of virulence [36]. By induction of FCZ resistance in *C. gattii* isolates, Santos et al., in 2014, showed that the virulence was diminished in a murine model in vivo [48]. However, the clinically resistant strain ST173 behaves differently since it is no less virulent than the susceptible strain. The acquisition of resistance to FCZ does not alter the virulence of the resistant clinical strain. There are no studies investigating the virulence of *C. gattii* strains clinically resistant to FCZ. Studies are focused on the virulence of strains made resistant through repeated exposure to FCZ. These results need to be confirmed with a larger number of FCZ-resistant strains and strains of different genotypes within the *C. gattii* species complex. To explain the differences observed in our work, it would be interesting to search and identify potential virulence factors that could explain and help to understand this different virulence phenotype.

## 5. Conclusions

Our study has shown that fluconazole-resistant clinical strains seem to differ from fluconazole induced resistant strains, both in terms of virulence and in terms of the molecular mechanisms of resistance. These results will, of course, have to be confirmed on a larger number of clinically resistant strains in order to explain the difference between the strains studied in this work.

## Data Availability

Not applicable.
